# Cutaneous Leishmaniasis and Conflict in Syria

**DOI:** 10.3201/eid2205.160042

**Published:** 2016-05

**Authors:** Waleed S. Al-Salem, David M. Pigott, Krishanthi Subramaniam, Lee Rafuse Haines, Louise Kelly-Hope, David H. Molyneux, Simon I. Hay, Alvaro Acosta-Serrano

**Affiliations:** Liverpool School of Tropical Medicine, Liverpool, UK (W.S. Al-Salem, K. Subramaniam, L.R. Haines, L. Kelly-Hope, D.H. Molyneux, A. Acosta-Serrano);; University of Oxford, Oxford, UK (D.M. Pigott, S.I. Hay);; University of Washington, Seattle, Washington, USA (S.I. Hay)

**Keywords:** Cutaneous leishmaniasis, sand fly, refugee camps, Syria, parasites, vector-borne infections, *Leishmania*, *Phlebotomus*

**To the Editor:** War, infection, and disease have always made intimate bedfellows, with disease recrudescence characterizing most conflict zones (*1*). Recently, increasing violence from civil war and terrorist activity in the Middle East has caused the largest human displacement in decades. A neglected consequence of this tragedy has been the reemergence of a cutaneous leishmaniasis epidemic.

Old World cutaneous leishmaniasis is one of the most prevalent insectborne diseases within the World Health Organization’s Eastern Mediterranean Region (*2*). Zoonotic cutaneous leishmaniasis is caused by the protozoan parasite *Leishmania major*, which is transmitted through the infectious bite of the female *Phlebotomus papatasi* sand fly; the animal reservoirs are the rodent genera *Rhombomys, Psammomys*, and *Meriones*. Anthroponotic cutaneous leishmaniasis is caused by *L. tropica* and transmitted between humans by the *Ph. sergenti* sand fly.

Until 1960, cutaneous leishmaniasis prevalence in Syria was restricted to 2 areas to which it is endemic (Aleppo and Damascus); preconflict (c. 2010) incidence was 23,000 cases/year (*3*). However, in early 2013, an alarming increase to 41,000 cutaneous leishmaniasis cases was reported (*3*,*4*). The regions most affected are under Islamic State control; 6,500 cases occurred in Ar-Raqqah, Diyar Al-Zour, and Hasakah. Because these places are not historical hotspots of cutaneous leishmaniasis, this change might be attributed to the massive human displacement within Syria and the ecologic disruption of sand fly (*Ph. papatasi*) habitats. According to the United Nations High Commissioner for Refugees, >4.2 million Syrians have been displaced into neighboring countries; Turkey, Lebanon, and Jordan have accepted most of these refugees. As a result, cutaneous leishmaniasis has begun to emerge in areas where displaced Syrians and disease reservoirs coexist (*5*).

According to the Lebanese Ministry of Health, during 2000–2012, only 6 cutaneous leishmaniasis cases were reported in Lebanon. However in 2013 alone, 1,033 new cases were reported, of which 96.6% occurred among the displaced Syrian refugee populations (*5*). Similarly in Turkey, nonendemic parasite strains *L. major* and *L. donovani* were introduced by incoming refugees (*6*).

Many of the temporary refugee settlements are predisposed to increased risk because of malnutrition, poor housing, absence of clean water, and inadequate sanitation. The combination of favorable climate, abundant sand fly populations, displaced refugees, and deficient medical facilities and services has created an environment conducive to cutaneous leishmaniasis reemergence. For example, refugee settlements in Nizip in southern Turkey have reported several hundred cases (*7*).

Using current datasets published in English and Arabic, we mapped cutaneous leishmaniasis prevalence within Syria and its neighboring countries (Figure). Our results demonstrate that cutaneous leishmaniasis prevalence coincides with the presence of refugee camps (Figure, panel A), which is plausible given the strong association between disease outbreaks and refugee settlements (*8*). The deterioration of Syrian health systems, including the cessation of countrywide vector control programs, has created an ideal environment for disease outbreaks (*9*). Likewise, the sand fly vectors are widely distributed throughout the Middle East; expansive *Ph. papatasi* and *Ph. sergenti* sand fly populations exist in Syria and Iraq (*4*). The presence of these vectors in regions of instability can create new cutaneous leishmaniasis foci, which might have debilitating, and often stigmatizing, consequences for residents and deployed military personnel (*10*). In addition, the distribution of *Leishmania* spp. overlaps with sand fly habitats (Figure, panel B) and disease reservoirs (W. Al-Salem, unpub. data). Consequently, the movement of large refugee populations into regions that are ill-equipped to manage imported cutaneous leishmaniasis has resulted in outbreaks in Turkey and Lebanon (*5*,*6*).

**Figure:**
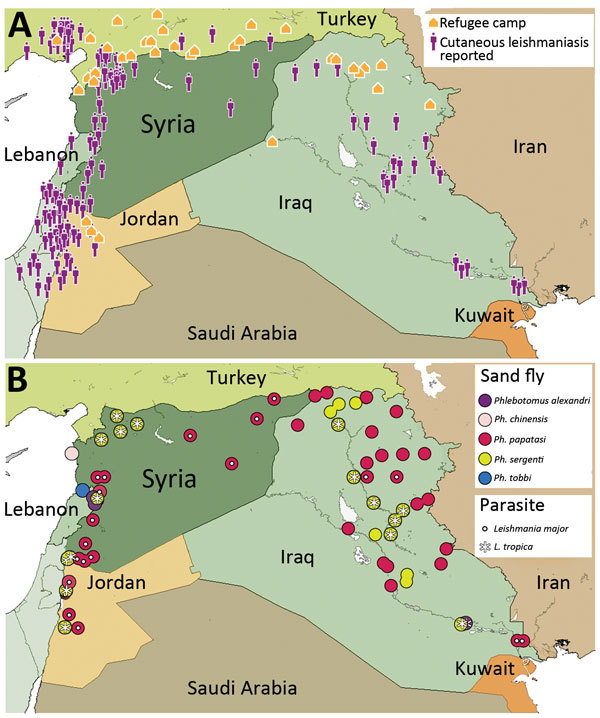
Cutaneous leishmaniasis prevalence within Syria and neighboring countries of the World Health Organization’s Eastern Mediterranean Region, 2013. A) Prevalence among refugee camps. Case data were taken from http://datadryad.org/resource/doi:10.5061/dryad.05f5h. B) Distribution of sand fly and parasite species. Country names and boundaries are not official. Maps were adapted from https://hiu.state.gov/Products/Syria_DisplacementRefugees_2015Apr17_HIU_U1214.pdf.

Our findings emphasize the importance of contemporaneous disease tracking to identify human populations at highest disease risk. To ameliorate the current cutaneous leishmaniasis crisis, particularly during the winter when cases start to appear, accurate disease monitoring and strategic training of persons based within refugee camps (medical staff, aid workers, volunteers, and military personnel) needs to be prioritized. Moreover, clinicians and other medical personnel residing in refugee-hosting countries must be suitably trained to diagnose cutaneous leishmaniasis because other local diseases (e.g., sarcoidosis and cutaneous tuberculosis) can have similar manifestations. Along with vector and rodent control, new cutaneous leishmaniasis outbreaks should be managed by prompt diagnosis and treatment, which are even more pertinent given that *L. tropica*–associated cutaneous leishmaniasis typically is resistant to several treatment regimens. In summary, the coexistence of sand fly populations and *Leishmania* spp. within refugee camps, together with the considerable influx of persons who already have cutaneous leishmaniasis, create a dangerous cocktail that can lead to an outbreak unprecedented in modern times.
